# Supplementary figures and images for: Senescent Myoblasts Exhibit ROS‐Dependent Akt‐mTORC1 Dysregulation and Are Susceptible to Reductive Stress‐Induced Cell Death
Source: Aging Cell. 2026 Jun 16;25(6):e70589. doi: 10.1111/acel.70589 (PMC13270776; doi:10.1111/acel.70589)

**A**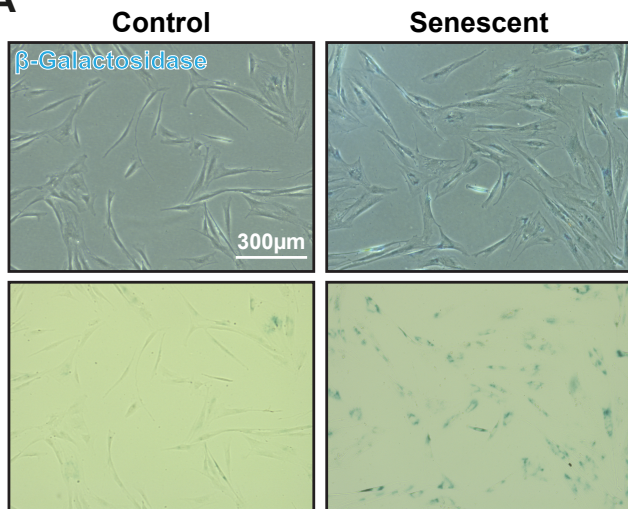**B**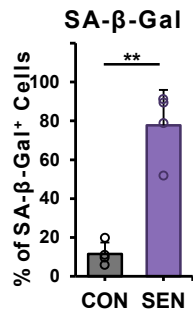**C**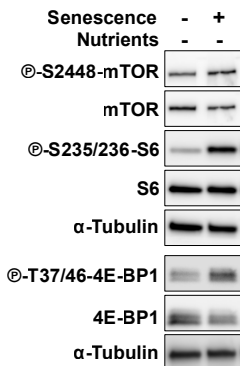**D**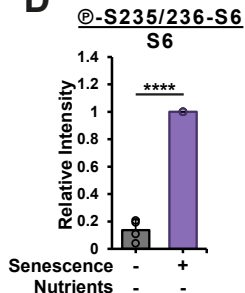**E**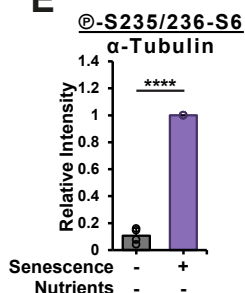**F**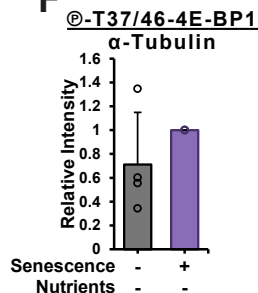**G**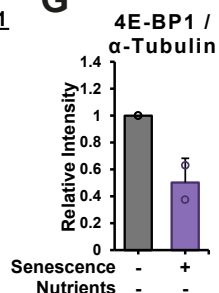

Supplement: Supplementary file 1 — Figure S1: mTORC1 dysregulation in primary human myoblasts. Primary human myoblasts were treated with 10 μM etoposide for 48 h and then maintained in growth medium for an additional 5 days (A, B) and starved in the last 19 h (C–G). (A, B) β‐galactosidase staining and quantification. (C–G) Immunoblot analysis with quantification of mTORC1 signalling markers. Results are reported as mean ± standard deviation. P values were calculated using independent, heteroscedastic t‐test. n = 4, except for G, where n = 2. **p ≤ 0.01, ****p ≤ 0.0001. CON, control; SEN, senescent. [file ACEL-25-e70589-s005.pdf]

**A**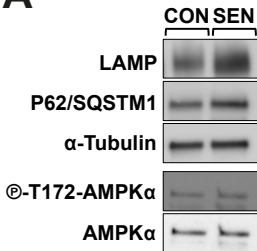**B**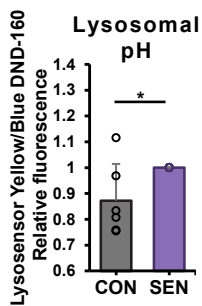**C**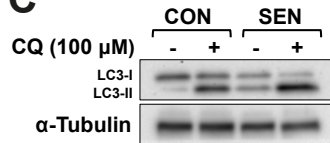**D**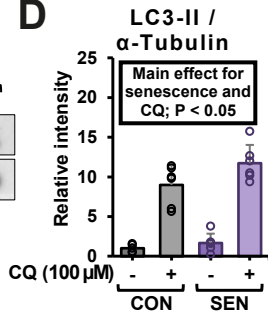**E**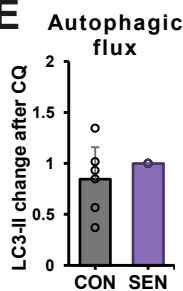**F**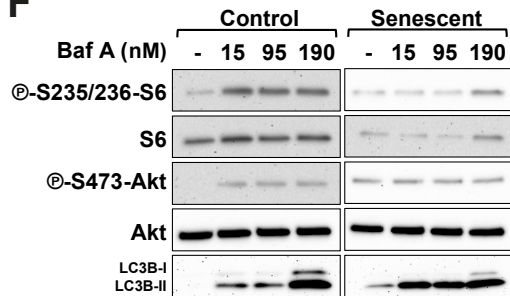**G**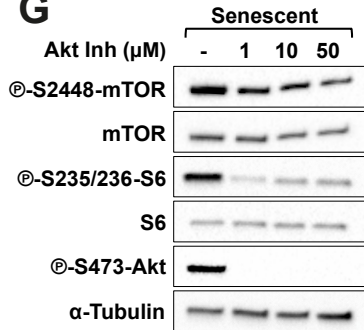**H**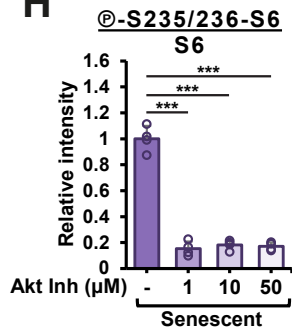

Supplement: Supplementary file 2 — Figure S2: Inhibition of Akt, but not autophagy, alleviates mTORC1 activity in starved senescent C2C12 myoblasts. (A, C, D and F–H) Immunoblot analysis and quantification for Akt/mTORC1 and autophagy‐related proteins in starved senescent C2C12 myoblasts treated with the indicated compound during the last hour of standard starvation protocol. (E) Autophagic flux calculation based on LC3‐II expression. (B) Lysosomal pH in non‐starved control and senescent myoblasts, measured using Lysosensor Yellow/Blue DND‐160. P values were calculated using an independent, heteroscedastic t‐test (B, E), one‐way ANOVA, (D and H) followed by Dunnett's post hoc test (H). n = 2 for (F) and 3–6 for the rest. *p ≤ 0.05, **p ≤ 0.01, ***p ≤ 0.001, ****p ≤ 0.0001. Akt Inh, Akt inhibitor; Baf A, bafilomycin A1; CON, control; CQ, chloroquine; SEN, senescent. [file ACEL-25-e70589-s006.pdf]

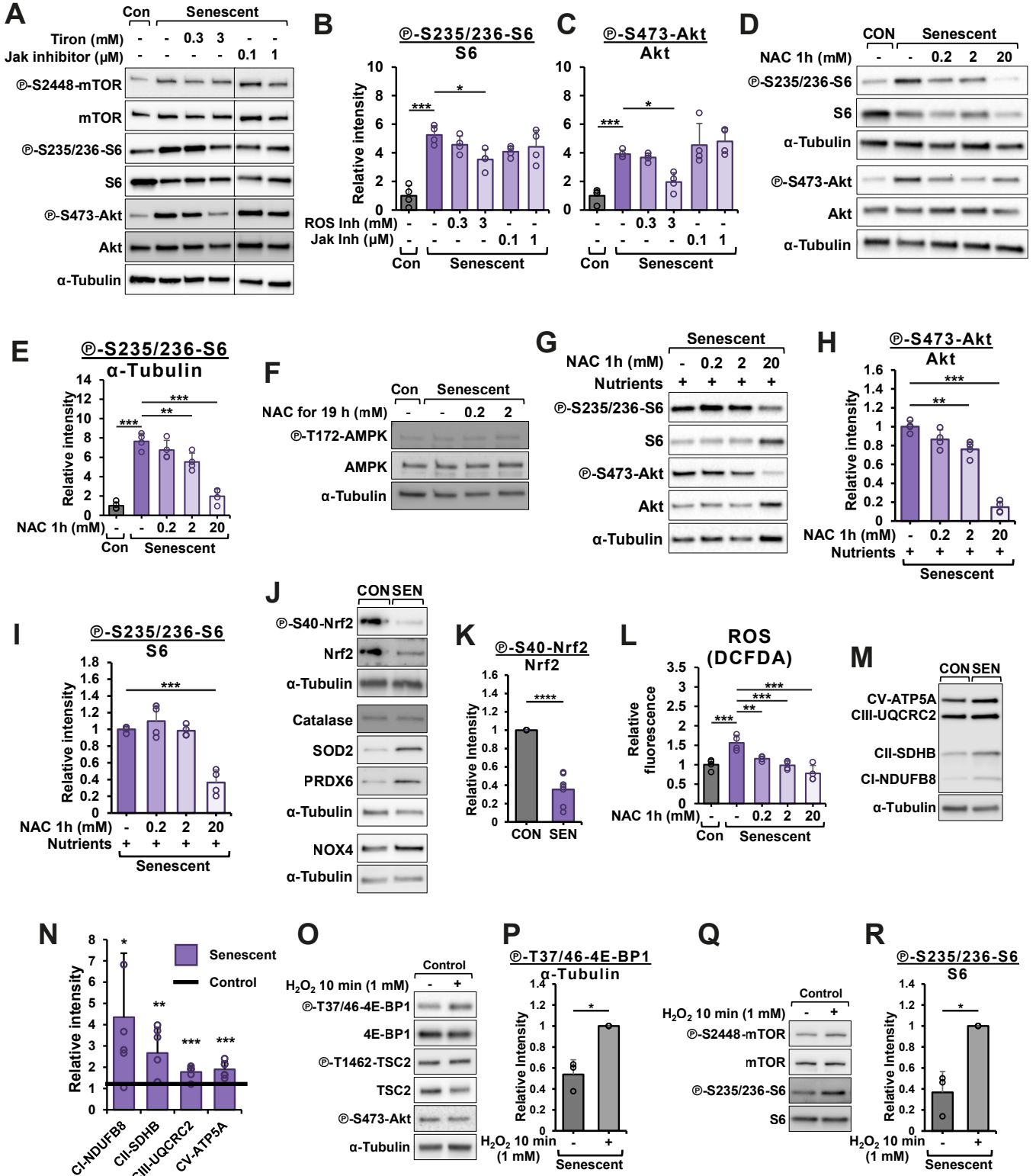

Supplement: Supplementary file 3 — Figure S3: Interaction between Akt/mTORC1 signalling and ROS. (A–C) Western blot analysis for Akt/mTORC1 signalling in starved senescent C2C12 myoblasts treated with the indicated doses of ROS scavenger/antioxidant tiron and Jak inhibitor tofacitinib. (D, E) Western blot analysis for Akt/mTORC1 signalling in starved senescent C2C12 myoblasts kept treated with indicated doses of NAC for 1 h. (F) Western blot analysis for AMPK signalling in starved senescent C2C12 myoblasts treated with the indicated doses of NAC for 19 h. (G–I) Immunoblot analysis and quantification for Akt/mTORC1 signalling in senescent C2C12 myoblasts kept in full GM treated with indicated doses of NAC for 1 h. (J, K) Western blot analysis of the transcriptional regulator of the antioxidant response (Nrf2, K), endogenous antioxidants (catalase, SOD2, PRDX6) and NOX4 in starved myoblasts. (L) DCFDA ROS assay quantified using a microplate reader in myoblasts treated with the indicated doses of NAC for 1 h. (M, N) Western blot analysis of ETC complexes in starved control and senescent cells. (O–R) Western blot analysis of Akt/mTORC1 signalling in starved control cells treated with H2O2. Results are reported as mean ± standard deviation. P values were calculated using an independent, heteroscedastic t‐test (K, N, P, R) and one way ANOVA followed by Dunnett's post hoc test (B, C, E, H, I, L). n = 3–7. *p ≤ 0.05, **p ≤ 0.01, ***p ≤ 0.001, ****p ≤ 0.0001. Con, control cells; Jak Inh = Jak inhibitor tofacitinib; NAC, N‐acetylcysteine; ROS Inh, ROS inhibitor/antioxidant Tiron. [file ACEL-25-e70589-s002.pdf]

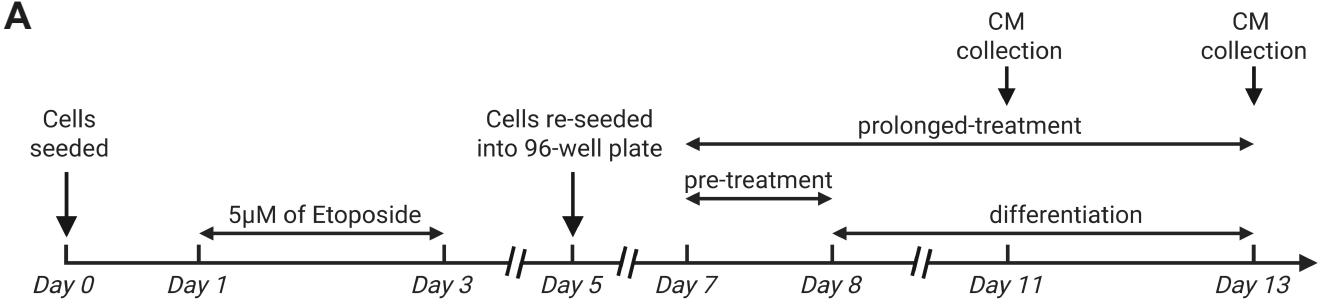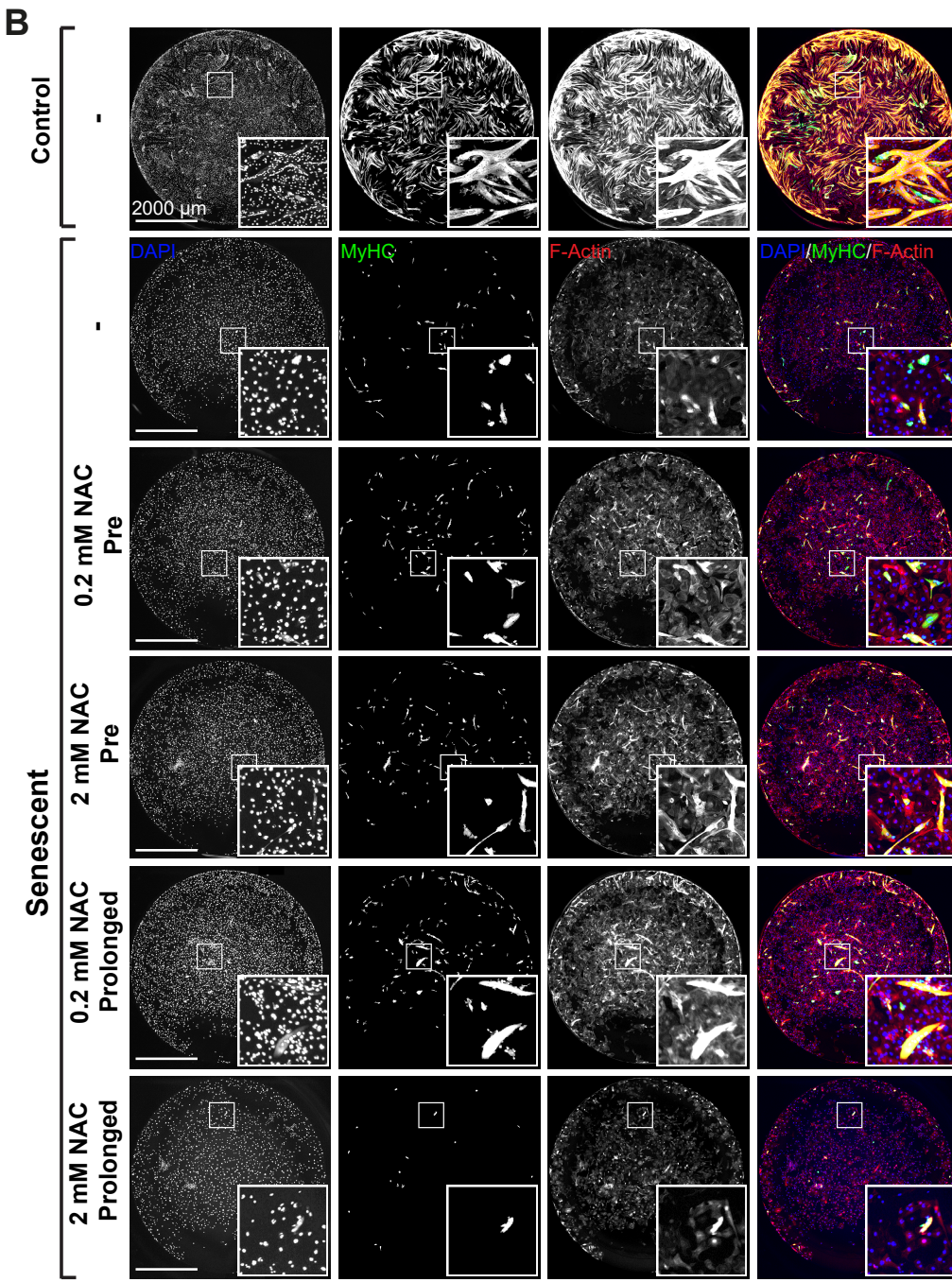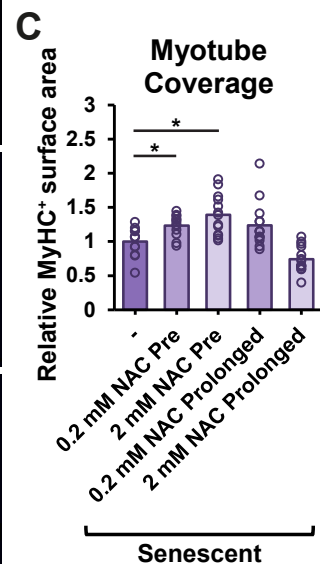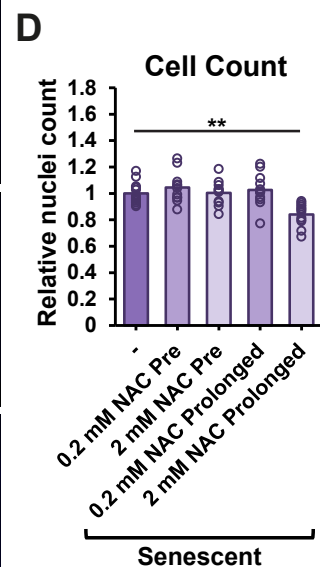

Supplement: Supplementary file 4 — Figure S4: The effect of antioxidants on myotube differentiation in senescent C2C12 myoblasts. (A) Protocol summary for all differentiation experiments. (B–D) Representative images (B) of differentiated senescent C2C12 myoblasts stained for F‐Actin and MyHC, and quantification of myotube coverage (C) and cell count (DAPI staining, D). Cells were stimulated to differentiate after 24 h pretreatment (Pre) or after 24 h pretreatment combined with concomitant antioxidant treatment during differentiation (Prolonged). Results are reported as mean ± standard deviation. P values were calculated using one way ANOVA followed by Dunnett's post hoc test. n = 9–12. *p ≤ 0.05, **p ≤ 0.01. CON, control; MitoQ, mitoquinone mesylate; NAC, N‐acetylcysteine; SEN, senescent. [file ACEL-25-e70589-s004.pdf]

**A**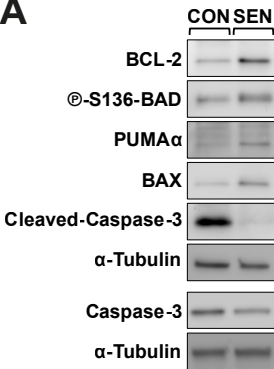**B**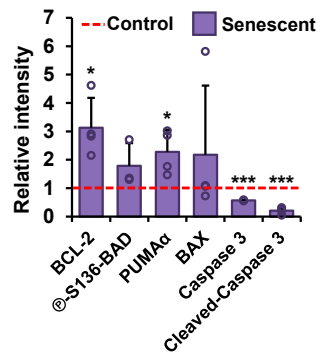**C**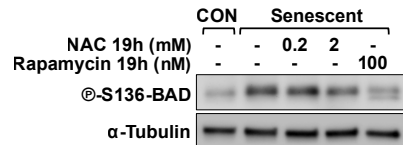**D**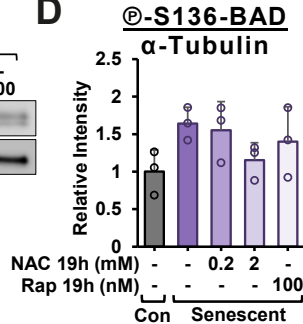**E**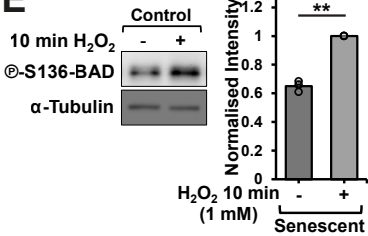**F**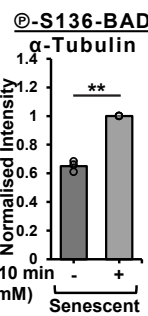**G**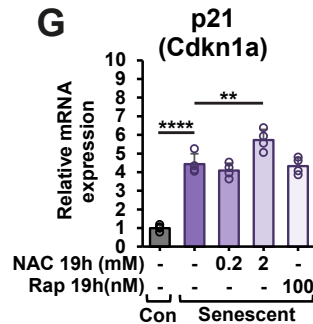**H**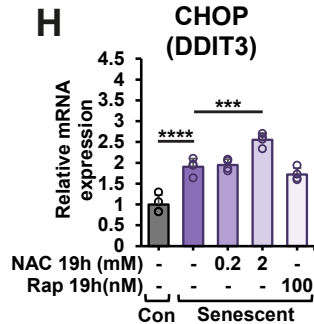**I**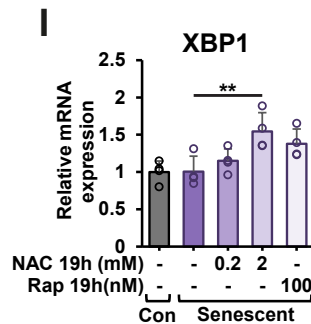

Supplement: Supplementary file 5 — Figure S5: Senescent C2C12 myoblasts are susceptible to antioxidant‐induced cell death. (A, B) Western blot analysis for cell survival related signalling in control and senescent myoblasts. (C–F) Western blot analysis of for phosphorylation of BAD in control and senescent myoblasts treated with NAC, rapamycin and H2O2 as indicated. (G–I) mRNA expression of DNA damage and ER stress‐related genes. Results are reported as mean ± standard deviation. P values were calculated using an independent, heteroscedastic t‐test (F), and one‐way ANOVA followed by Dunnett's post hoc test (the rest). *p ≤ 0.05, **p ≤ 0.01, ***p ≤ 0.001, ****p ≤ 0.0001. CON, control; NAC, N‐acetylcysteine, Rap, rapamycin; SEN, senescent. [file ACEL-25-e70589-s001.pdf]
